# Extubation failure in intensive care unit: Predictors and management

**DOI:** 10.4103/0972-5229.40942

**Published:** 2008

**Authors:** Atul P. Kulkarni, Vandana Agarwal

**Affiliations:** **From:** Department of Anesthesiology, Critical Care and Pain, Tata Memorial Hospital, Parel, Mumbai, Maharashtra, India

**Keywords:** Extubation, failure of, predictors of, reintubation, weaning

## Abstract

Extubation failure-need for reintubation within 72 h of extubation, is common in intensive care unit (ICU). It can cause increased morbidity, higher costs, higher ICU and hospital length of stay (LOS) and mortality. Patients with advanced age, high severity of illness at ICU admission and extubation, preexisting chronic respiratory and cardiovascular disorders are at increased risk of extubation failure. Unresolved illness, development and progression of organ failure during the time from extubation to reintubation and reintubation itself have been proposed as reasons for increased morbidity and mortality. Parameters used to predict extubation failure can be categorized into parameters assessing respiratory mechanics, airway patency and protection and cardiovascular reserve. Adequate cough strength, minimal secretions and alertness are necessary for successful extubation. Evidence suggests that early institution of non-invasive ventilation and prophylactic administration of methylprednisolone may prevent reintubation in some patients. The intensivist needs to identify patients at high risk of extubation failure and be prepared to reinstitute ventilation early to prevent adverse outcomes.

## Introduction

After resolution of illness, mechanically ventilated patients are liberated from the ventilator, the process being called weaning. It is essential to understand that weaning and extubation though following each other in clinical practice, are two separate processes which pose distinct problems. Extubation failure is defined as inability to sustain spontaneous breathing after removal of the artificial airway; an endotracheal tube or tracheostomy tube; and need for reintubation within a specified time period: either within 24-72 h[1,2] or up to 7 days.[3,4] Substantial literature exists about weaning predictors and outcomes; most being inaccurate in predicting extubation outcome. To predict “extubation failure” is essential, as both delayed and failed extubation have detrimental consequences such as prolonged ventilation and ICU stay, need for tracheostomy, increased cost of treatment and mortality.[5–7] In this review, we discuss causes, outcomes, predictors and management of extubation failure in patients passing spontaneous breathing trial (SBT).

## Incidence and Consequences of Extubation Failure

Incidence of extubation failure [Table 1] varies between 6 and 47%.[3,8,9] Epstein[3] and Gowardman[10] found that patients needing reintubation had significantly increased duration of mechanical ventilation as well as ICU and hospital LOS and mortality. The OUTCOMERA study group[11] found a significant increase in the incidence of nosocomial pneumonia with consequent increase in the duration of mechanical ventilation, hospital and ICU LOS, without increased mortality. In a case control study,[7] the incidence of pneumonia was significantly higher (47% vs. 10%) in patients needing reintubation. Dries and colleagues[12] also found an increased incidence of nosocomial pneumonia in patients who failed extubation. The reported[3,5] mortality rates in patients with extubation failure vary between 30 and 40%.

**Table 1:** Incidence and causes of failed extubation studies are listed as per year of publication

**Author/year (No. of falled/total extubations) (%)**	Reasons for reintubation (number of patients)*								
	
	**Upper-airway obstruction**	**Impaired clearance of secretions**	**Respiratory failure**	**Hypoxemia**	**Hypercapnia**	**Insecure airway**	**Cardiac Failure**	**Neurological impairment**	**Others**
Lee[17] 1994 (9/52) 17%		1	3				1	2	3
Capdevila[16] 1995 (12/67) 18%			8	3			1		
Miller[8] 1996 (17/100) 6%	3	5#	8				3		1
Daley[13] (24/405) 7%			7			5		12	5
Epstein[5] (74)**	11	12	21				17	7	6
Esteban[56] 1999 (61/526) 11.5%	9	17 + 5#	23	20	7		4	11	8
Rady[15] 1999 (748) 6.6%	60		122	406		60	153	7	
Coplin[23] 2000 (24/136) 18%			13^a^						11^b^
Khamiees[40] 2001 (18/91) 20%		4	9	4	2		6^c^	3	
Smina[1] 2003 (13/95) 13.6%	2			3	2	5	1^d^		
Martinez[2] 2003 (10/69) 14.4%		1		9					
Salam[20] (10/69) 14.4%		1		12				4	1
Esteban[52] 2004 (106/221) 47%		11	48	24	11		6	6	
jiang[38] 2004 (11/55) 20%		4	6				4	3	
Naval[53] 2005 (16/97) 16%	2	5		2			2	2	3
Gowardman[10] 2006 (52/2761) 1.8%	10	17	12			7	4	2
Robriquet[28] 2006 (52/148) 35%	2	14		16	18		2		
Frutos-Vivar[22] 2006 (121/900) 13.4%		12	55	27	13		7	7^e^
Hernandz[34] 2007 (19/93) 20%		4	3	3	4		1^d^	3	1
Mokhlesi[21] 2007 (16/122) 13%	2	3	2	1			2	2	1^f^ + 3^g^

* Some points had more than one reason of failure

** All patients had failed extubation

# atelectasis

a combination of respiratory failure and airway causes

b unplanned surgery and other medical problems

c HR ≥ 120 bpm

d cardiac arrest

e hypotension

f unclear reason

g progression of the underlying process

## Extubation Failure and Adverse Outcomes: Pathophysiology

Deconditioned muscles, poor nutrition, upper airway edema due to prolonged translaryngeal intubation, inability to clear secretions, decreased level of consciousness due to persistent effects of sedative and analgesics and critical illness polyneuropathy; all can lead to extubation failure. Several hypotheses attempt to explain increased mortality associated with failed extubation. One hypothesis is that increased mortality after extubation failure may reflect a sicker cohort of patients,[3] with failed extubation acting as an additional marker of severity of illness. This derives from the fact that extubation failure retains a strong independent effect on mortality even after adjusting for confounders such as generalized severity of illness, chronic comorbidities, age and need for acute dialysis. Second hypothesis is that reintubation, an invasive procedure, may itself increase mortality. This may be due to the life-threatening events during reintubation such as cardiac arrest, esophageal intubation, endobronchial intubation, aspiration of gastric contents and cardiac arrhythmias. Torres and colleagues[7] found that patients requiring reintubation were more likely to develop nosocomial pneumonia (47% vs. 10%) with increased mortality. However, if reintubation itself caused mortality, then mortality should not vary for different causes of extubation failure. Esteban and colleagues[4] found a lower mortality for patients reintubated for upper airway obstruction than those reintubated for respiratory failure (7% vs. 30%). This is because reintubation for upper airway obstruction rapidly corrects respiratory dysfunction; whereas organ dysfunction related to other causes of extubation failure may not be readily reversible. Mortality, however, was not different in trauma patients[13] needing reintubation for stridor, inability to clear secretions or deteriorating sensorium. Third hypothesis is that clinical deterioration occurs during spontaneous breathing with development of new organ dysfunction, thereby increasing mortality. This may explain in part the relationship between cause of extubation failure and outcome. This hypothesis is further supported by increase in mortality with increasing duration of time between extubation and reintubation, independent of etiology of extubation failure, mortality for respiratory failure increased four times when reintubation occurred >12 h after extubation.[5] Another, similar hypothesis presumes a relationship between delay in reintubation and increased mortality. This delay allows a progressive clinical deterioration in the patients' condition leading to organ dysfunction/failure. Though not confirmed in a recent Australian study,[10] several other studies[5,9,14] have reported an increase in mortality with increased time to reintubation beyond 12-24 h.

## Risk Factors for Extubation Failure

The risk factors for failed extubation vary depending on the population studied. Rady and colleagues[15] reported older age, preexisting left ventricular dysfunction, anemia, large transfusion requirements and renal dysfunction to be the risk factors for extubation failure in cardiac surgical population. Older age,[16] severity of illness on ICU admission, prolonged duration of ventilation before extubation[17] and the use of continuous sedation[18] have been identified as risk factors for extubation failure. Neurologic impairment was found to be an independent risk factor for extubation failure in several studies.[19–21] Pre-extubation hypercapnoea (PCO_2_ ≥ 44 mmHg) due to an imbalance of respiratory load and capacity was an independent risk factor for extubation failure.[21] Frutos-Vivar[22] found that positive fluid balance in preceding 24 h led to extubation failure. Salam and colleagues[20] found that the predictive accuracy for failed extubation increased to 100% when neurologic status was considered along with adequacy of cough peak flows and volume of endotracheal secretions. In brain injured patients, Coplin and colleagues[23] found no correlation between GCS score and reintubation.

## Causes of Extubation Failure

Table 1 lists the common causes of extubation failure, some being common to a failed spontaneous breathing trial (SBT). Causes related to airway patency and secretion may manifest only after extubation. The most common cause is respiratory failure which manifests with increased work of breathing, accessory muscle use, hypoxia and/or hypercapnea and respiratory acidosis. Another frequent reason is failure to maintain airway patency due to upper airway edema, seen in patients with prolonged translaryngeal intubation and evident as stridor. Excessive secretions, coupled with inadequate muscle strength and glottic incompetence can also cause failed extubation. Neurological impairment, due to primary illness, encephalopathy or residual effect of sedatives can necessitate reintubation. Although the most frequent cause of weaning failure is respiratory system failure, a possible consequence of the transfer from assisted to spontaneous breathing can be acute myocardial ischemia or cardiac dysfunction/failure.[24] The clinical signs presented by patients who fail a weaning trial because they develop acute heart failure (e.g., tachypnoea, tachycardia, anxiety) are often difficult to distinguish from clinical signs resulting from respiratory system failure. The time course of B-type natriuretic peptide serum concentration[25] can help to differentiate between respiratory and cardiac etiology.

## Predictors of Failed Extubation

A recent metaanalysis[26] found that commonly used predictors such as respiratory mechanics performed moderately when predicting successful SBT and but these predictors did even worse when extubation failure alone was studied. This may be due to the differences in pathophysiology of weaning and extubation failure. While weaning predictors identify the imbalance between respiratory capacity and load,[27] extubation failure can occur due to other causes as well. It may also be that the clinician has assessed these factors intuitively, before conducting SBT. Decisions regarding the timing of extubation are based on assessment of the patient's capacity to protect the airway, quantity of secretions and cough strength. In this review we discuss predictors of failed extubation in patients who have successfully completed SBT.

## Demographic Predictors of Extubation Failure

Numerous demographic predictors have been described. Rady and colleagues,[15] in patients undergoing cardiovascular surgery, found that extubation failure was significantly more likely to occur in patients with: old age, preoperative comorbidities, need for preoperative intra-aortic balloon pump and multiple transfusions (>10 units), surgery of thoracic aorta and prolonged cardiopulmonary bypass. They suggested that patients with these factors may be excluded from routine “fast track” extubation protocol. Similar comorbidities may cause extubation failure in other ICU patients. Other demographic predictors are severity of illness at ICU admission (high APACHE II) and prolonged duration of ventilation.[17,26] A recent trial[28] in COPD patients found that SAPS II > 35 at ICU admission and need for home ventilation predicted extubation failure. Pneumonia as reason for mechanical ventilation with unresolved infection predicted extubation failure.[22]

## Predictors Assessing Respiratory Mechanics

Most parameters of respiratory mechanics are useful only for predicting successful SBT and perform moderately or poorly with extubation prediction. We therefore present a brief discussion of these parameters.

### Rapid shallow breathing index (RSBI - f/V_T_)

Capdevila[16] found significantly lower values of f/V_T_ (50 ± 23 vs. 69 ± 25 breaths/min/l) in successfully extubated patients. Recently a cut off value of RSBI ≥ 57 was described[22] to separate patients who could not be extubated successfully. Epstein,[27] however, found that with f/V_T_ < 100, 14 (of 84) patients failed extubation, 13 due to other organ system problems and suggested that f/V_T_ was not physiologically suited to predict extubation failure. Other studies[2,20,29] have also reported inability of f/V_T_ for predicting extubation outcome.

### Airway occlusion pressure at 1 s (P_0.1_) and Ratio of occlusion pressure to maximum inspiratory pressure (MIP) [P_0.1_/MIP]

P_0.1_/MIP is an index of balance between respiratory reserve and demand and reflects neuromuscular drive for breathing and it is unaffected by respiratory compliance or resistance. Capdevila and colleagues[16] reported that patients with low P_0.1_ and P_0.1_/MIP failed extubation, Mergoni and colleagues[30] reported excellent prediction of success in weaning using P_0.1_/MIP, while Del Rosario[31] found similar P_0.1_/MIP values in patients with weaning success and failure. In a metaanalysis,[26] P_0.1_/MIP ratio of >0.3 had a pooled likelihood ratio of 2.3, indicating increased chances of successful extubation. Despite excellent predictive accuracy, the role of P_0.1_/MIP ratio may be limited in most ICUs due to need for a special device.

### Minute ventilation recovery time (V_E_ RT)

Minute ventilation recovery time (V_E_RT) allows physiologic assessment of work imposed after SBT. Thus V_E_RT may identify patients with better respiratory muscle reserve, capable of sustaining spontaneous breathing following extubation. Martinez and colleagues,[2] after a 2-h SBT, placed patients back on their pre-SBT ventilator settings for up to 25 min and measured various respiratory parameters including minute ventilation (V_E_) at three intervals: baseline over preceding 24 h (pre-SBT), post-trial (after SBT) and recovery (return to baseline). Patients were assumed to recover when V_E_ decreased to 110% of the predetermined baseline. V_E_RT of patients with successful extubation was significantly shorter than those who failed extubation (3.6 ± 2.7 min vs. 9.6 ± 5.8 min, *P* < 0.011). On multivariate analysis, V_E_RT was an independent predictor of extubation outcome and correlated with ICU LOS (*P* < 0.01). Prolonged V_E_RT may reflect either a limited respiratory reserve or an unrecognized, underlying disease process. Seymour and colleagues[32] evaluated a more practical method. For pre-SBT V_E_ they collected data in three ways, a 24-h nadir (as in previous study), an 8-h average and the last V_E_ measurement prior to SBT. They also collected data on V_E_RT with threshold of 100% and 110%. They found that both, the 8-h average V_E_ and immediate pre-SBT value of V_E,_ were in close agreement with the original method. Similarly 100% threshold for V_E_RT also correlated well with 110% threshold. The same group later demonstrated[33] the utility of the new method in predicting extubation success. Recently Hernandez and colleagues[34] evaluated the utility of close observation of V_E_ during the recovery phase after the SBT. Both V_E_RT RT50% ΔV_E_ (recovery time needed to reduce V_E_ to half the difference between End-SBT- V_E_ and basal V_E_) were significantly lower in successfully extubated patients. They found that a threshold of RT50% ΔV_E_ of seven minutes was useful to discriminate between extubation failures and successes. V_E_ RT and derivatives appear to be promising tools, the drawback being their inability to identify patients with possible upper airway compromise.

### Work of breathing (WOB)

Kirton and others[35] found that patients who fail SBT due to increased imposed work of breathing (WOB) secondary to ventilatory apparatus and endotracheal tube, but have normal physiological WOB, can be successfully extubated. The same group later showed[36] that when physiological WOB ≤0.8 J/l, patients can be successfully extubated. Automatic Tube Compensation (ATC), a form of variable pressure support, was shown[37] to improve extubation success by reducing imposed work of breathing. WOB, a promising predictor; remains confined to the research setting due to technical difficulties.

### Displacement of liver/spleen

Diaphragm fatigue results in slower movement and reduced excursion. Jiang and colleagues[38] hypothesized that liver and spleen displacement during SBT can be a surrogate of diaphragmatic endurance. In 55 ICU patients, two separate observers measured the displacement of liver and spleen by ultrasonography. Patients were extubated by clinicians blinded to the study results. Patients who were successfully extubated had higher mean values. With a cutoff value of 1.1 cm, the sensitivity and specificity to predict successful extubation was 84.4% and 82.6%. This is a noninvasive test and can be done bedside, but needs expertise.

## Parameters Assessing Airway Protection

### Cough strength, peak expiratory flow and airway secretions

Adequate cough strength, dependent on the ability of respiratory muscles to generate pressure and flows, is important to clear airway secretions; the so-called “airway competence”. This may be compromised due to unresolved pulmonary pathology, weak respiratory muscles and laryngeal dysfunction and compounded by increased resistive work due to airway inflammation and bronchospasm. Secretions may be increased due to irritation by endotracheal tube, non-infectious inflammation, lower or upper respiratory tract infection or aspiration of secretions.

Bach and Saporito[39] found that in tracheostomized patients assisted peak cough flows (PCF) were greater in those who were successfully decannulated. Khamiees and colleagues[40] evaluated the cough strength on command (0 to 5) and amount of endotracheal secretions (none to abundant) in patients passing SBT. Patients were then asked to cough onto a white card through the endotracheal tube. If secretions were propelled onto the card, it was termed a positive white card test (WCT). Patients with weak (grade 0 to 2) coughs and abundant secretions were more likely to fail extubation. Negative WCT also predicted failed extubation. In another study[20] PCF, volume of endotracheal secretions and neurologic status were evaluated after successful SBT. Patients with low PCF failed extubation and a threshold of CPF 60 l/min was a useful discriminator. Impaired neurologic status and secretion volume of >2.5 ml/h increased the risk of extubation failure. With three risk factors 100% patients failed extubation as against 3% without any risk factors. A recent study[21] found moderate to copious secretions to be an independent predictor of extubation failure. The same group,[1] later found that patients with PEF <60 l/min were likely to fail extubation and die in the hospital. The peak flows in these studies were much lower than Bach's study[39] (60 l/min vs. 160 l/min) as intubated patients cannot close their glottis, limiting the pressure and flow generation. Measured cough strength is objective, inexpensive and can be done easily at the bedside and appears to be useful in predicting extubation outcome. However, this test requires patient cooperation. If the patient fails to comprehend instructions, this can lead to erroneous results, reflecting lack of cooperation rather than insufficient muscle strength. Coplin and colleagues[23] evaluated brain injured patients using a semi-quantitative airway care score (ACS) comprising six parts and assigned four points each: spontaneous cough, gag, sputum quantity, sputum viscosity, suctioning frequency and sputum character. ACS did not correlate with either success or failure of extubation. Eighty nine per cent patients with absent or weak gag and 82% patients with weak or absent cough were successfully extubated. However, they found that adequate spontaneous cough and low suctioning frequency were associated with successful extubation. Namen and colleagues[19] also did not find any relationship of presence of cough reflex and cough during suctioning and extubation failure.

### Neurological dysfunction

Good mentation is essential for airway protection. The rate of reintubation was highest (33%) in patients with neurologic disease in a trial[41] examining clinical characteristics of patients undergoing weaning. In brain injured patients,[23] 24 of 136 patients required reintubation, but none due to neurological impairment. 80% of patients with GCS ≤ 8 and 91% of patients with GCS ≤ 4 were successfully extubated. There was no correlation between GCS and need for reintubation. 44 (of 117) neurosurgical patients[19] failed extubation and 22 patients needed reintubation. Patients with successful extubation had higher GCS score. A GCS ≥ 8 showed highest predictive accuracy with further improvement with increasing GCS. GCS ≥ 10 has been suggested recently[21] to be a prerequisite for successful extubation.

## Parameters Assessing Airway Patency

### Cuff leak test

Postextubation laryngeal edema and airway obstruction frequently leads to failed extubation. Adderley and Mullins[42] using “Qualitative cuff leak test” during a croup epidemic, found that 38% patients with absence of leak, required reintubation. The premise is that with endotracheal tube cuff deflated air leak will occur. The amount of leak depends on the degree of laryngeal and airway edema and complete absence of leak indicates very severe edema. Miller and Cole[8] used “Quantitative Cuff leak test” - average cuff leak volume during inspiration and expiration. A cuff leak volume of < 110 ml, predicted a significant risk of postextubation stridor. “Quantitative Cuff leak test” is reproducible and objective. Other studies have used similar[43] or higher[44] threshold values of cuff leak volume. Jaber and colleagues[44] used a cut-off threshold of leak 12% of expired tidal volume; while De Bast and colleagues[45] used leak threshold 15.5% of expired tidal volume. Secretions encrusted on the outer part of the endotracheal tube may reduce cuff leak volume and confound the results. It is also important to understand that though the cuff leak test predicts occurrence of post extubation stridor, it cannot predict the need for reintubation.

### Laryngeal ultrasound

Ding and colleagues[46] performed a quantitative cuff leak test and bronchoscopy after a 30-min SBT. Real-time ultrasonography (US) was also done to evaluate the air-leak and the air-column width with the endotracheal cuff inflated and deflated. The air column width during cuff deflation was significantly lower in patients who developed post extubation stridor.

It is a noninvasive, reliable method, but requires skilled expert to perform the procedure.

## Parameters Assessing Hemodynamics and Tissue Perfusion

Change from assisted to spontaneous breathing during weaning acts as cardiovascular stress test. Patients with normal cardiac reserve increase cardiac output to meet this increased demand while patients with diminished cardiac reserve increase O_2_ extraction.[47] Most predictors have been assessed with a view to weaning rather than extubation success. Grasso and colleagues[25] found evidence of acute left ventricular dysfunction in patients who failed weaning. Gastric mucosal CO_2_ is a surrogate of intramucosal pH and increase in Gastric - arterial CO_2_ difference (ΔPg-aCO_2_) beyond 10 mmHg indicates inadequate splanchnic blood flow.[48] Uusaro and colleagues[29] evaluated change in ΔPg-aCO_2_ to predict extubation failure in patients with successful SBT. After stress test (spontaneous breathing at 0 pressure support), the ΔPg-aCO_2_ was significantly higher in 17 patients (25%) who failed extubation. 30 patients out of 31 with ΔPg-aCO_2_ < 12 mmHg were successfully extubated. ΔPg-aCO_2_ after stress seems to be a very good predictor of likelihood of successful extubation. Patients with positive fluid balance in 24 h preceding extubation were reported[22] to need reintubation more frequently. This may represent persistent vascular permeability due to unresolved illness, however, since hemodynamic data are reported, cardiovascular insufficiency with pulmonary edema cannot be ruled out.

## Management of Failed Extubation

A reasonable strategy to prevent failed extubation, if anticipated, can be continued ventilation, treatment of remediable causes of muscle weakness and excessive secretions and daily assessment for readiness to extubate, until predictors become more favorable. Delayed extubation may lead to several complications like pneumonia, increased ICU and hospital LOS, increased cost and mortality.[23] Specific therapies can be used only when the cause for failed extubation is known. If extubation failure is due to cardiac failure, adequate anti-failure with diuretics and vasodilators can be instituted and then extubation attempted. Performing tracheostomy is another option; however, the problem of removing artificial airway still remains and tracheostomy has its own complications. Only two options seem promising in failed extubation, non-invasive ventilation and prophylactic steroids.

### Non-invasive ventilation

Non-invasive ventilation (NIV) is used in acute exacerbation of COPD and to prevent intubation and ventilation. It seems logical that NIV may avert reintubation after failed extubation as well. Several studies[49–52] have evaluated use of NIV, with mixed results. Nava and colleagues[53] found that application of NIV immediately after extubation led to reduced reintubation rates and ICU mortality and reintubation was a strong predictor of mortality. After a prospective trial, Ferrer and colleagues[18] reported NIV to be useful for preventing reintubation and reducing mortality only in patients with hypercapnea during SBT. They suggested that NIV is likely to be useful for patients with COPD and other chronic respiratory disorders, but not in general ICU population, similar to NIV use in acute exacerbation of COPD.[54] NIV seems to be effective when used early in patients with COPD and in those who are hypercapnic during SBT.

### Role of steroids

Extubation failure caused by upper airway edema is difficult to assess before extubation. Cuff leak test predicts stridor but not need for reintubation. Prophylactic use of steroids reduced reintubation rate in high risk neonates and children but not in low risk pediatric patients.[55] A Cochrane review,[56] first published in 1999 and reviewed again in 2004, showed no benefit of prophylactic steroids in adults. In a recent double blinded trial,[57] patients planned for extubation received four (20 mg) doses of methylprednisolone starting 12 h before extubation at 4h intervals or placebo. Prophylactic methylprednisolone reduced (from 22 to 3%) incidence of laryngeal edema and rate of reintubation due to (8% vs. 54%) laryngeal edema. After prolonged intubation, patients should undergo a quantitative cuff leak test when ready to be weaned. If positive, they should receive prophylactic methylprednisolone to prevent reintubation.

## Conclusion

Extubation failure is common in ICU and leads to increased morbidity, costs and mortality. Good mentation, competent airway, minimal secretions, good respiratory muscle strength and adequate cardiovascular reserve are essential for successful extubation. Combination of predictors may predict extubation failure accurately[20] but the results remain to be duplicated. We need to assess therapies to improve respiratory muscle strength, laryngeal competence, neurological status and secretion load and also whether these therapies improve extubation success. Simple, noninvasive predictor of cardiovascular reserve is needed, apart from other predictors. Till such predictors can be validated in general ICU population, one needs to be alert for extubation failure and intervene early to prevent further morbidity.
